# The great East Japan earthquake affected the laboratory findings of hemodialysis patients in Fukushima

**DOI:** 10.1186/1471-2369-14-239

**Published:** 2013-10-30

**Authors:** Nobuhiro Haga, Junya Hata, Michihiro Yabe, Kei Ishibashi, Norio Takahashi, Ken Kumagai, Souichiro Ogawa, Masao Kataoka, Hidenori Akaihata, Yoshiyuki Kojima

**Affiliations:** 1 Department of Urology, Fukushima Medical University School of Medicine, Fukushima, Japan 1 Hikarigaoka, Fukushima 960-1295, Japan; 2 Department of Urology, Soma Central Hospital, Fukushima, Japan; 3 Department of Urology, Hanawa Welfare Hospital, Fukushima, Japan

**Keywords:** Laboratory findings, Cardiothoracic ratio, Hemodialysis, Earthquake, End-stage renal disease

## Abstract

**Background:**

The aim of the present study was to investigate the impact of the Great East Japan Earthquake on laboratory findings in chronic hemodialysis (HD) patients in Fukushima.

**Methods:**

Changes in laboratory findings and cardiothoracic ratio (CTR) between before and after the earthquake were retrospectively analyzed in 90 adult HD patients with end-stage renal disease (ESRD). Two hospitals located within 80 km from the Fukushima Daiichi Nuclear Power Plant, where American government recommended to evacuate from the area, participated in the study. HD duration was shortened by 0.5-1 hour for 1 month after the earthquake. Multivariate analyses were performed to identify the factors contributing to change of measurement values.

**Results:**

Blood urea nitrogen (BUN) value was significantly transiently decreased for 1-2 weeks after the earthquake (*P*=0.002). In multivariate analysis, age showed a tendency to be related to the decrease of BUN level (*P*=0.05). Hematocrit value was significantly elevated after two months compared with that at baseline (*P*=0.02), although the elevation was small. The other measured values and CTR were not significantly changed compared with those before the earthquake.

**Conclusions:**

Laboratory findings and CTR did not worsen despite the shortening of HD duration. Hence, in this disaster, as far as chronic HD patients with ESRD were concerned, it was possible for the duration of HD treatment to be safely decreased.

## Background

The Great East Japan Earthquake, measuring 9.0 on the Richter scale, struck Fukushima prefecture on March 11, 2011. About 20,000 people died or disappeared due to the direct effects of trauma by the earthquake as well as a huge tsunami that subsequently occurred. In Fukushima prefecture, secondary damage associated with this disaster occurred due to an accident at Fukushima Daiichi Nuclear Power Plant, which was a Level-7 nuclear event, defined as the most serious on the International Nuclear and Radiological Event Scale (INES). Several explosions and other events led to the scattering of a large amount of radioactive substances into the air, water, and soil [1]. Residents living within 20 km of Fukushima Daiichi Nuclear Power Plant were ordered to evacuate immediately after the accident.

After the disaster, infrastructure, such as roads, bridges, rail, water pipes, and telecommunication systems, was severely damaged. In addition, the flow of various supplies, such as food, water, medicine, and gasoline, was interrupted. The main causes of this interruption were that the infrastructure was severely damaged and that individuals tasked with transporting such supplies were not willing to enter Fukushima prefecture because of fears about the possibility of radiation exposure associated with the nuclear accident. Moreover, supplies could not be gathered directly from other prefectures owing to a shortage of gasoline. As such, there was the potential for an increase in physically or psychologically adverse effects for not only healthy people but also patients with chronic conditions like end-stage renal disease (ESRD) requiring hemodialysis (HD) in the affected area. Residents in the area were compelled to consume relief food for days or weeks as a result of this situation. Relief food contains high potassium levels or high salt levels to preserve it against decay [2,3]. While food restriction is very important for chronic HD patients with ESRD, consumption of relief food might led to hyperkalemia or volume overload, causing fatal complications [4]. However, the limited availability of food in Fukushima prefecture could have led to these problems.

Although the impact of a major disaster on HD patients with ESRD has been investigated in several studies [5-8], an analysis based on laboratory findings has not been reported. In the present study, we investigated the impact of the earthquake on the laboratory findings of HD patients with ESRD.

## Methods

### Study population

**Figure 1:**
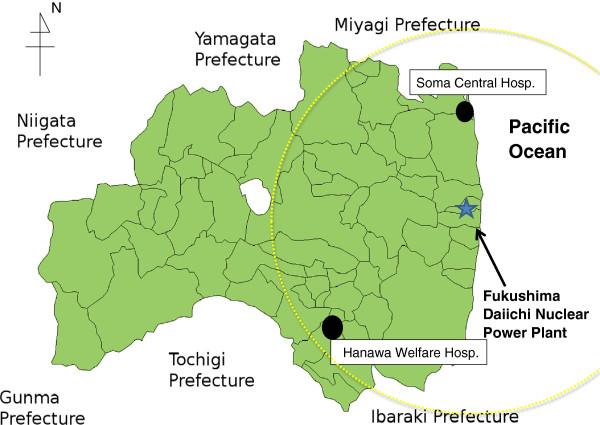
**Map of the Fukushima Daiichi nuclear power plant and two hospitals located in Fukushima prefecture.** Yellow dotted circle represents the nuclear evacuation zone recommended by American government.

This study included 90 adult HD patients with ESRD who had been on maintenance HD three times weekly (4 hours per session) for at least 3 months before the earthquake in Fukushima prefecture. Changes in laboratory data, cardiothoracic ratio (CTR) on chest radiographs and interdialitic weight gain before and after the earthquake were retrospectively analyzed. Two hospitals participated in this study. One is Soma Central Hospital, located 2 km away from the coast, which suffered from serious damage from the huge tsunami that resulted from the earthquake, which took the lives of 475 people living in Soma city. In addition, Soma city is located 40 km away from Fukushima Daiichi Nuclear Power Plant. The other hospital is Hanawa Welfare Hospital, which was not damaged by the tsunami and is located 70 km away from Fukushima Daiichi Nuclear Power Plant. However, both hospitals were located within 80 km from the Fukushima Daiichi Nuclear Power Plant, where American government recommended to evacuate from the area (Figure 1). Then, the flow of various supplies, such as food, water, medicine, and gasoline, was interrupted. Both hospitals were partially destroyed, but the hemodialysis (HD) procedures could be continued immediately after the earthquake without missed dialysis sessions. In Soma Central Hospital, three patients who were killed by the huge tsunami and one patient who needed to be evacuated from their home after the earthquake were excluded from the study. Diabetes mellitus was defined by glucose values ≥ 200 mg/dl at any time, fasting glucose values ≥ 126 mg/dl, or the use of insulin or oral hypoglycemic drugs. Hypertension was defined as a clinical systolic blood pressure (BP) of at least 140 mmHg or a diastolic BP of at least 90 mmHg measured on two or more occasions or the use of antihypertensive medications.

This study complied with the Declaration of Helsinki and was approved by the ethics committee of Hanawa Welfare Hospital and the ethics committee of Soma Central Hospital. All participants read and signed an informed consent form prior to this investigation.

### Dialysis procedures

The overall number of dialysis machines is 44 (Soma hospital; 18, Hanawa; 36). All patients underwent HD three times weekly (4 hours per session), using 1.2-2.1 m^2^ surface area dialyzers with bicarbonate-based dialysates (sodium, 140 mEq/L (mmol/L); bicarbonate, 30 mEq/L (mmol/L); potassium, 2.0 mEq/L (mmol/L); calcium, 3.0 mEq/L; and magnesium, 1.0 mEq/L). The blood flow rate was 150-200 ml/min. The dialysate flow rate was 500 ml/min. The dialysate temperature was 36.5°C. The mean value of Kt/V before the earthquake was 1.01±0.16. However, soon after the earthquake, our department, which was composed of some board-certified specialists for dialysis, rapidly decided to reduce the HD duration at both our affiliated hospitals. Because we anticipated the difficulties in saving the dialysate and tap water, moreover, both hospitals received the hemodialysis patients from other hospitals and performed hemodialysis for them, we decided to shorten dialysis duration.

HD duration was shortened by 1 hour for about 2 weeks, and was then shortened by 0.5 hours for the next 2 weeks because of incomplete recovery from damage to the infrastructure, and thus both hospitals had low reserves of water, dialysates, medicines, and so on.

### Clinical parameter measurement

Blood samples were drawn before and after HD. Blood urea nitrogen (BUN), creatinine, sodium, potassium, chloride, hemoglobin, hematocrit (Hct), fasting blood glucose (FBS), and hemoglobinA_1c_ (HbA_1c_) concentration were analyzed. FBS and HbA_1c_ were tested only for the patients diagnosed with diabetes mellitus.

CTR was measured on chest radiographs. Standard chest radiographs were taken in a standing position via the posterior-anterior view. CTR was determined by dividing the maximal horizontal width of the heart by the horizontal inner width of the rib cage.

Blood examination results and CTR from just before the earthquake were picked up as baseline data. After the earthquake, measurement values in the first/second week (first examination), and those in the fourth week (second examination), and the eighth week (third examination) were picked up as post-earthquake data.

### Statistical analysis

All values are presented as mean ± standard deviation or median. Time-dependent change of values was statistically analyzed using one-way analysis of variance with the Bonferroni post hoc test. Two-sided Mann-Whitney *U* test was used to determine significant differences of values in binary variables. The correlations between measurement values and continuous variables were investigated by simple regression analysis using the Spearman rank correlation coefficient. Multivariate analyses were performed using multiple linear regression to identify the factors contributing to change of measurement values. *P*-values < 0.05 were considered significant. Analyses were performed with StatView version 5.0 software (Abacus Concepts, Berkeley, CA, USA).

## Results

The baseline characteristics of the patients are shown in Table 1. BUN value was significantly decreased at the first examination (1-2 weeks after the earthquake) (*P*=0.002) (Table 2). However, it returned to baseline after the second examination (4 weeks after the earthquake) (Table 2). Univariate and multivariate analyses were performed to investigate the factors contributing to the decrease of BUN level. In univariate analysis, female sex (*P*=0.04) and increasing age (*P*=0.008) were significantly associated with the decrease of BUN level (Table 3a, 3b). In multivariate analysis, increasing age tended to be related to the decrease of BUN level (*P*=0.05) (Table 3c). In addition, Hct value was significantly elevated at the third examination (8weekes after the earthquake) compared with that at baseline (*P*=0.02), although the elevation was small (Table 2). Univariate analyses were performed to investigate the factors contributing to the increase of Hct level. In univariate analysis, the difference of hospital tended to be related to the increase of Hct level (*P*=0.05) (Table 4). Multivariate analyses were not performed with regard to Hct because unvariate analyses did not achieve the statistical significance. The other measurement values, interdialytic weight gain, and CTR were not significantly changed compared with those before the earthquake (Table 2). Up to 2-month follow-up period, we could not find the increased disaster-related mortality and morbidity caused by the anorexia or food restriction.

**Table 1:** Patient characteristics at baseline

**Variables**	
**N**	**90**
Age (years)	66 (36–87)
	58: 32
Sex (male: female)	54.3
Diabetes	30 (33.3%)
Hypertension	66 (73%)
Hypertensive medications	
RAS inhibitor	45 (50%)
Ca channel blocker	49 (54%)
Sympatholytic	32 (35%)
Hospital (Hanawa: Soma)	52: 38

RAS inhibitor includes angiotensin II receptor blocker, angiotensin-converting enzyme inhibitor, and direct renin inhibitor.

Sympatholytic includes a-blocker, b-blocker, and central sympatholytic. Hanawa; Hanawa Welfare Hospital, Soma; Soma Central Hospital.

**Table 2:** Changes clinical data of hemodialysis patients before and after the earthquake

	**Baseline**	**First exam.**	***P***	**Second exam.**	***P***	**Tthird exam.**	***P***
	***Before earthquake***	***1–2 weeks***		***4 weeks***		***8 weeks***	
	**Mean±SD**	**Mean±SD**		**Mean±SD**		**Mean±SD**	
***Biochemical data***							
**Predialysis BUN (mg/dl)**	60.7±13.8	53.5±14.1*	0.002	57.3±15.7	0.15	61.2±16.2	0.10
**Post dialysis BUN (mg/dl)**	19.5±5.8	17.4±4.3	0.23	19.8±6.1	0.75	18.6±5.7	0.78
**Creatinine (mg/dl)**	10.9±3.0	10.9±3.1	0.98	11.0±3.2	0.76	10.7±2.9	0.69
**Sodium (mEq/l)**	139.2±3.6	139.4±2.7	0.84	139.3±3.2	0.78	138.6±5.6	0.34
**Potassium (mEq/l)**	4.6±0.6	4,5±0.6	0.23	4.5±0.7	0.42	4.5±0.6	0.20
**Chlorine (mEq/l)**	103.3±3.4	102.7±5.0	0.70	103.9±5.0	0.29	101.5±7.7	0.07
**Hemoglobin (g/dl)**	11.0±1.0	11.1±1.0	0.67	11.0±0.8	0.89	11.0±1.1	0.94
**Hematocrit (%)**	33.2±3.0	33.0±4.3	0.74	34.0±3.0	0.11	34.4 ± 3.5*	0.02
**FBS (mg/dl)**	158±66	155±64	0.85	151±66	0.65	170 ± 69	0.44
**HbA1c (%)**	7.0±1.3	**-**	**-**	6.7±1.4	0.61	6.8±1.3	0.69
**Change of DW (Kg)**	2.2±0.8	2.2±1.0	0.58	2.0±0.8	0.18	2.0±0.8	0.32
**CTR (%)**	49.7±6.2	49.2±6.1	0.63	50.5 ± 6.5	0.41	50.3±6.3	0.54

*BUN*, blood urea nitrogen; *FBS*, fasting blood glucose; *HbA*_*1c*_, hemoglobin-A_1c_ concentration; *DW*, dry weight; *CTR*, cardiothoracic ratio; *P<0.05 vs. baseline values.

Baseline means previous data from before the earthquake. First exam., Second exam., and Third exam. are the examinations 1-2 weeks, 4 weeks, and 8 weeks after the earthquake, respectively.

**Table 3:** Univariate and multivariate analyses of various factors in relation to a reduction of BUN level 1 week after the earthquake

**a) BUN according to patient characteristics using Mann-Whitney *U *** **test**			
		**BUN**	
		**Mean±SD**	***P***
**Sex**			0.04*
	Male	55.7±15.0	
	Female	49.1±11.2	
**Diabetes**			**0.74**
	Yes	54.2±15.3	
	No	53.1±13.5	
**Hypertension**			**0.09**
	Yes	52.0±13.8	
	No	58.0±14.4	
**Hospital**			**0.22**
	Hanawa	55.0±14.1	
	Soma	51.1±14.1	
**b) Correlation between BUN and patient characteristics using simple regression analysis**			
		**BUN**	
		***C.C***	***P***
**Age**		0.29	0.008*
**DW**		0.17	**0.12**
**Height**		0.20	**0.09**
**c) Multivariate analysis of various factors in relation to reduction of BUN level 1 week after the earthquake**			
		**BUN**	
		**b**	***P***
**Sex**		0.12	**0.28**
**Diabetes**		0.02	**0.82**
**Hospital**		−0.12	**0.27**
**Age**		−0.23	**0.05**

*BUN*, blood urea nitrogen; *SD*, standard deviation; Hanawa, Hanawa Welfare Hospital, not seriously damaged by the tsunami and far from the nuclear power plant accident;

Soma, Soma Central Hospital, seriously damaged by the tsunami and near the nuclear power plant accident; *c.c.*, correlation coefficient; *DW*, dry weight; b, standard partial regression coefficient; * p<0.05.

**Table 4:** Univariate analyses of various factors in relation to a reduction of Hct level 8 week after the earthquake

**a) Hct according to patient characteristics using Mann-Whitney *U *** **test**			
		**HCT**	
		**Mean±SD**	***P***
**Sex**			**0.71**
	Male	34.3±3.7	
	Female	34.6±3.3	
**Diabetes**			**0.57**
	Yes	34.1±4.0	
	No	34.5±3.3	
**Hypertension**			**0.86**
	Yes	34.4±3.6	
	No	34.2±3.2	
**Hospital**			**0.05**
	Hanawa	35.0±4.0	
	Soma	33.4±4.0	
**b) Correlation between Hct and patient characteristics using simple linear regression analysis**			
		**Hct**	
		***C.C***	***P***
**Age**		0.17	**0.13**
**DW**		0.06	**0.57**
**Height**		0.08	**0.49**
**Change of DW**		0.05	**0.63**

*Hct*, hematocrit; *SD*, standard deviation; Hanawa, Hanawa Welfare Hospital, not seriously damaged by the tsunami and far from the nuclear power plant accident;

Soma, Soma Central Hospital, seriously damaged by the tsunami and near the nuclear power plant accident; *c.c.*, correlation coefficient; *DW*, dry weight; b, standard partial regression coefficient; * p<0.05.

## Discussion

A catastrophic earthquake might lead to morbidity and mortality among chronic HD patients with ESRD, not only by the direct impact of the earthquake but also by interfering with the HD due to infrastructural damage and shortages of medicine and disposable HD items [4,9,10]. With regard to the hospitals that participated in the current study, both hospitals’ buildings were partially damaged and the reverse osmosis (RO) system was damaged at Soma Central Hospital. However, maintenance HD was continued immediately after the Great East Japan Earthquake at both hospitals, but, for about one month, the duration of HD was shortened by 0.5-1 hour owing to a shortage of materials for the HD treatment.

In the present study, mean value of Kt/V before the disaster was 1.01±0.16. In large multicenter study in Japan, the mean value of Kt/V was 1.31±0.3 [11]. Then, providing the Kt/V of at least 1.28 might further improve survival in Japanese patients who had undergone hemodialysis for more than 10 years [12]. In the present study, the mean value of Kt/V before the disaster did not reach that level. Hence, laboratory follow-up was needed soon after the disaster, because the reducing dialysis duration might affect the patients’ condition in the present study, compared with well dialysed patients with high Kt/V.

After a catastrophic earthquake, laboratory follow-up of HD patients is difficult because of damage to equipment, shortage of laboratory chemicals, and absence of laboratory technicians [9]. Hence, to our knowledge, this is the first study to describe the laboratory findings in HD patients with ESRD after a major earthquake.

The present study demonstrated that BUN level was significantly decreased during the first to second week (the first examination) after the earthquake. This finding suggests that transient anorexia or food restriction in chronic HD patients might have occurred, although Inui *et al.* reported that, after a catastrophic earthquake, dietary intake in patients with anorexia nervosa did not necessarily decrease [13]. Then, we investigated the factors contributing to the decrease of BUN level. Increasing age showed a tendency to be related to the decrease of BUN level in multivariate analysis (*P*=0.05). Additionally, although we selected two hospitals with different levels of damage due to the tsunami and nuclear power accident, we did not show that the resultant differences in psychological stress might have led to differences in the BUN level in HD patients.

Hct level was significantly elevated two months after the earthquake (the third examination) in the current study. The difference of hospital tended to be related to the increase of Hct level. However, as the elevation was small, it might not constitute clinically meaningful data.

In the patients with diabetes mellitus at the Great Hanshin-Awaji Earthquake in 1995 in Japan, HbA_1c_ concentration was increased after the earthquake compared with that before it owing to chronic psychological stress [14]. However, the present study did not demonstrate that FBS and HbA_1c_ concentration changed after the earthquake. The reason for this discrepancy might be that, in the present study, we investigated ESRD patients with diabetes mellitus and the observation period was shorter in the present study than in the above-mentioned study.

In the Great Hanshin-Awaji Earthquake, a significant increase of CTR was transiently observed in chronic HD patients who were in the seriously damaged area during and after the earthquake [15]. In the present study, although the duration of HD was shortened, no increase of CTR and interdialytic weight gain was observed. Additionally, aggravation of biochemical data, such as hyperkalemia, was not observed. These results including for BUN level can be accounted for by increased compliance of patients with dietary and fluid restrictions, probably due to worry about not being able to receive dialysis treatment in the coming days [9] and a good relationship between medical staff and patients [16]. These data suggest that the duration of HD could be safely reduced, at least for a limited period. In the Marmara Earthquake in Turkey, Sever *et al.* reported that the frequency of HD treatment could be safely reduced, although the laboratory findings were not investigated [9]. Indeed, well dialysed patients with very high Kt/V might have a “reserve” allowing them to decrease dialysis time for a few weeks-months with careful attention to laboratory finding and body weight gain.

Several limitations of the present study must be considered. First, this study included a small sample size. This may have limited the power to find associations between patient characteristics and change of laboratory findings and CTR. Second, we did not investigate every laboratory finding because of the shortage of laboratory chemicals. Third, we did not investigate the laboratory findings and CTR within 1 week after the disaster because the necessary equipment was not fully prepared. However, in Fukushima prefecture, in particular at the “exposed” hospital, this period might have been the most critical because of the shortage of supplies and the physical and psychological stress associated with the fear of nuclear explosions and earthquake-related events, such as another huge tsunami being triggered by the frequent aftershocks, as well as death in the family and the loss of property induced by the earlier tsunami.

## Conclusions

In the present study, laboratory findings and CTR were not exacerbated despite a shortening of HD duration. Hence, in this disaster, as far as chronic HD patients were concerned, it is possible that the duration of HD treatment could be safely decreased.

## Abbreviations

INES: International nuclear and radiological event scale; ESRD: End-stage renal disease; HD: Hemodialysis; CTR: Cardiothoracic ratio; BP: Blood pressure; BUN: Blood urea nitrogen; Hct: Hematocrit.

## Competing interest

We declare that we have no conflicts of interest.

## Authors’ contributions

NH, MK and HA carried out the studies of clinical trials and drafted the manuscript. HJ and MH participated in collecting and organizing of data. KI and NT performed the statistical analysis. KU, SO and YK conceived of the study, and participated in its design and coordination and helped to draft the manuscript. All authors read and approved the final manuscript.

## Pre-publication history

The pre-publication history for this paper can be accessed here:

http://www.biomedcentral.com/1471-2369/14/239/prepub
